# A thank you to DMM's peer reviewers

**DOI:** 10.1242/dmm.039313

**Published:** 2019-02-28

**Authors:** Rachel Hackett

**Affiliations:** Rachel Hackett is the Managing Editor of DMM; Disease Models & Mechanisms, The Company of Biologists, Bidder Building, Station Road, Cambridge CB24 9LF, UK

## Abstract

The staff and editors of Disease Models & Mechanisms (DMM) wish to thank all our peer reviewers for their work in this vital role. We highlight some important changes that have been introduced to improve the peer-review process, for both authors and reviewers.

## Abstract (precis)

**Summary:** DMM highlights changes in our peer-review process, and thanks peer reviewers for their involvement in this vital step in scholarly publication.

## Cross-referee commenting

Peer review is the vital cornerstone of scholarly publication. Disease Models & Mechanisms (DMM) continuously aims to improve the day-to-day experience of our hundreds of peer reviewers annually, and occasionally this involves strategic decisions from the editor team. As a result of our discussions, DMM adopted a more collaborative form of peer review in July 2018. DMM peer reviewers now have a 48-h window during which they are invited to comment on the other referee reports before the editor makes a decision. The aim of this ‘cross-referee commenting’ step is to help resolve differences between referees, identify unnecessary or unreasonable requests, or – conversely – highlight valid concerns raised by one referee but overlooked by others. Six months later and at least 20% of papers have at least one reviewer having provided cross-referee comments. We hope to see uptake increase as reviewers become more familiar with the process.

## Early-career scientists as co-reviewers

DMM encourages the involvement of postdocs and other early-career scientists in the peer-review process. We ask that the name of the co-reviewer is reported to the Handling Editor, and a field is now provided in the peer-review report form for this purpose. Mentoring early-career scientists in the art of peer review is crucial to the future of scientific publishing; judging by the number of names recorded in the DMM co-reviewer field, it is an opportunity that many of our directly invited reviewers take. The names of all our 2018 reviewers, including, for the first time, their co-reviewers, are listed in the supplementary material. We publicly thank them all for devoting their time and expertise to review for DMM; we absolutely couldn't do it without you.

## Supplementary Material

Supplementary information

