# Supplementary material for: A thank you to DMM's peer reviewers
Source: Dis Model Mech. 2019 Feb 28;12(2):dmm039313. doi: 10.1242/dmm.039313 (PMC6398495; doi:10.1242/dmm.039313)
Supplement: Supplementary information [file dmm-12-039313-s1.pdf]

Reviewers for Disease Models & Mechanisms 2018

Sarah Abdul-Wajid, University California San Francisco, USA
Usha Acharya, University of Massachusetts Medical School, USA
James Adjaye, Heinrich Heine University Düsseldorf, Germany
Imran Ahmad, The Beatson Institute for Cancer Research, UK
Timothy Aitman, University of Edinburgh, UK
Stephen Alexander, University of Missouri-Columbia, USA
Fowzan Alkuraya, King Faisal Specialist Hospital and Research Center, Saudi Arabia
Rachel Allison, Mississipi State University, USA
Yolanda Almaden Peña, IMIBIC, Reina Sofia University Hospital, Spain
James Amatruda, UT Southwestern Medical Center, USA
James Amos-Landgraf, University of Missouri, USA
María Amparo Lago Huville, UNSM - National University of General San Martin, Argentina
Matt Anderson, University of Wyoming, USA
Helder André, Karolinska Institute, Sweden
Victor Anggono, University of Queensland, Australia
Lynda Aoudjehane, Human HepCell - UPMC, France
Samuel Aparicio, University of British Columbia, Canada
Suneel Apte, Cleveland Clinic Lerner Research Institute, USA
Atsushi Asakura, Stem Cell Institute, University of Minnesota, USA
Hirotaka Ata, Mayo Clinic, USA
Magda Atilano, University College London, UK
Paul August, Icagen, USA
Aboobaker Aziz, University of Oxford, UK
Nadine Aziz, Boston University School of Medicine, USA
Ken Baker, Cleveland Clinic Lerner Research Institute, USA
Jeroen Bakkers, Hubrecht Institute, Netherlands
Elisa Bal de Kier Joffe, University of Buenos Aires, Argentina
Tamas Balla, NIH, Bethesda, USA
Kathryn Bambino, Icahn School of Medicine Mount Sinai, USA
Simon Bamforth, Newcastle University, UK
Thomas Baranski, Washington University, USA
Barbara Bardoni, CNRS, France
Isabelle Baro, L'Institut du Thorax, University of Nantes, France
Marisa Bartolomei, The Pearleman School of Medicine, University of Pennsylvania, USA
Madhumita Basu, Virginia Commonwealth University, USA
Jean-François Beaulieu, Université de Sherbrooke, Canada
Thomas Becker, University of Edinburgh, UK
Aaron Beedle, SUNY Binghamton University, USA
Christian Beetz, Jena University Hospital, Germany
David Beier, University of Washington, USA
Hugo Bellen, Baylor College of Medicine, USA
Melina Bellin, Leiden University Medical Center, The Netherlands
Paola Bellosta, University of Trento, Italy
Fahad Benthani, Memorial Sloan Kettering Cancer Center, USA
Jason Berman, Dalhousie University, Canada
Monique Bernsen, Erasmus MC, Rotterdam, The Netherlands
Roberta Besio, University of Pavia, Italy
Ilya Bezprozvanny, UT Southwestern Medical Center, USA
Shipra Bhatia, The University of Edinburgh, UK
Marta Biagioli, University of Trento, Italy
Colin Bingle, The University of Sheffield Medical School, UK
Thomas Bird, Universtiy of Edinburgh, UK
Serge Birman, CNRS, ESPCI ParisTech, France
Kivanc Birsoy, Rockefeller University, USA
Ashis Kumer Biswas, University of Colorado Denver, USA
Karen Blyth, The Beatson Institute for Cancer Research, UK
Rolf Bodmer, Sanford Burnham Prebys Medical Discovery Institute, USA
Graeme Bolger, University of Alabama at Birmingham, USA

Disease Models & Mechanisms • Supplementary information

Andrea Bonetto, Indiana University, USA
Carolin Bonner, Institut Pasteur de Lille, France
Joseph V. Bonventre, Harvard Stem Cell Institute, USA
Cesario Borlongan, University of South Florida, USA
Alexander Borowsky, UC Davis Cancer Center, USA
Luke Boulter, University of Edinburgh, UK
Teresa Bowman, Albert Einstein College of Medicine, USA
Michael Boylan, Marymount University, USA
Steven Bradfute, University of New Mexico, USA
Andrea Brancaccio, ICRM, CNR Università Cattolica del Sacro Cuore, Italy
Thomas Braulke, University Medical Center Hamburg-Eppendorf, Germany
David Brenner, University of California San Diego, USA
Molly Brewer, UConn Health, USA
Garrett Brinkley, University of Alabama at Birmingham, USA
Steven Brody, Washington University School of Medicine, USA
Elizabeth Brooks, Duke University Medical Center, USA
Marco Brotto, University of Texas, Arlington, USA
Anthony Brown, Weill Cornell Medical College, USA
Liam Brunham, University of British Columbia, Canada
Valerie Brunton, University of Edinburgh, UK
Andrew Bryant, University of Florida Health, USA
Robert Bryson-Richardson, Monash University, Australia
Timothy Bullock, University of Virginia, USA
Alexa Burger, University of Zurich, Institute of Molecular Life Sciences, Switzerland
Harold Burgess, National Institute of Child Health and Human Development, USA
Ezra Burstein, UT Southwestern Medical Center, USA
Sharon Byers, University of Adelaide, Australia
Ross Cagan, Icahn School of Medicine at Mount Sinai, USA
Dana Cairns, Tufts University, USA
Nigel Calcutt, University of California San Diego, USA
Peter Campbell, Wellcome Sanger Institute, UK
Marina Campione, CNR Institute of Neurosciences, Italy
Loredana Capobianco, University of Salento, Italy
Pere-Joan Cardona, Fundació Institut "Germans Trias i Pujol" (IGTP), Spain
Christopher Cardozo, Icahn School of Medicine at Mount Sinai, USA
Rita Carini, University of Piemonte Orientale, Italy
Carlos Carmona-Fontaine, New York University, USA
Thomas Carroll, UT Southwestern Medical Center, USA
Tamara Caspary, Emory University, USA
Pierre Cattenoz, Institut de Génétique et de Biologie Moléculaire et Cellulaire, France
Anil Kumar Challa, University of Alabama at Birmingham, USA
Danny Chan, The University of Hong Kong, Hong Kong
Ho Yin Edwin Chan, Chinese University of Hong Kong, China
Hugh Chan, Cleveland Clinic Lerner Research Institute, USA
Uma Chandrachud, Harvard Medical School, Boston, USA
Krish Chandrasekaran, University of Maryland, Baltimore, USA
Catalina Chang, Northwestern University, USA
Pamela Chang, Cornell University, USA
Jau-Nian Chen, University of California, Los Angeles, USA
Wenbiao Chen, Vanderbilt University School of Medicine, USA
Xiao Chen, Massachusetts Institute of Technology, USA
Nikki Cheng, University of Kansas Medical Center, USA
Yu-Shan Cheng, NIH, Bethesda, USA
Sarah Childs, University of Calgary, Canada
Nitin Chitranshi, Macquarie University, Australia
Se-Young Choung, Kyung Hee University, Democratic People's Republic of Korea
Jyoti Chuckowree, University of Tasmania, Australia
Yuen-Li Chung, The Institute of Cancer Research, UK
Steven Clapcote, University of Leeds, UK
Michael Clarke, Stanford Medicine, USA

Susanne Clee, University of British Columbia, Canada
David Clouthier, University of Colorado, Denver, USA
Phil Coan, The University of Edinburgh, UK
Jörn Coers, Duke University School of Medicine, USA
Struan Coleman, The Hospital for Special Surgery, USA
Keon Colett, Dalhousie University, USA
Candice Contet, The Scripps Research Institute, USA
Matthew Cook, Australian National University, Australia
David Cooper, University of Alabama at Birmingham, USA
Gabriele Corda, University of Oxford, UK
Fernando Corrales, Centro Nacional de Biotecnología, Spain
Susan Cotman, Massachusetts General Hospital, USA
Rachel Cox, Uniformed Services University, USA
Roger Cox, Medical Research Council Harwell, UK
Timothy Cox, University of Cambridge, UK
Mark Cronan, Duke University School of Medicine, USA
Peter Crouch, University of Melbourne, Australia
Veronika Csizmok, Michael Smith Laboratories Vancouver, USA
Vincent Cunliffe, University of Sheffield, UK
Christine Curcio, University of Alabama School of Medicine, USA
Ales Cvekl, Albert Einstein College of Medicine, USA
Marija Cvetanovic, University of Minnesota, USA
Tim Czopka, Technical University of Munich, Germany
James Dachtler, University of Durham, UK
Maria-Antonietta D'Agostino, Centre Hôpital Ambroise Paré, France
Gokhan Dalgin, The University of Chicago, USA
Radbod Darabi, University of Texas, Houston, USA
Maria Elena de Bellard, California State University, Northridge, USA
Pietro De Camilli, Yale University, USA
Danilo De Gregorio, McGill University, Canada
Susana De la Luna, Centre for Genomic Regulation (CRG), Spain
Qing Deng, Purdue University, USA
Donna Denton, University of South Australia, Australia
Evandro De-Souza, Universidade Federal do Rio de Janeiro, Brazil
Danelle Devenport, Princeton University, USA
Kelly Dew-Budd, University of Arizona, USA
María Inés Diaz Bessone, UNSM - National University of General San Martin, Argentina
Mary Dickinson, Baylor College of Medicine, USA
Paula Dietrich, University of Tennessee Health Science Center, USA
Jeff Dilworth, University of Ottawa, Canada
Lee Dolat, Duke University School of Medicine, USA
James Dowling, Hospital for Sick Children, Canada
Ioannis Dragatsis, University of Tennessee, USA
Zdenek Drahota, Institute of Physiology (IPHYS), Czech Republic
Yigal Dror, University of Toronto, Canada
Michael Duchen, University College London, UK
Carrie Duckworth, The University of Liverpool, UK
Janet Duerr, Ohio University, USA
Brian Eames, University of Saskatchewan, Canada
Kate Eaton, University of Michigan, USA
Gunter Eckert, Justus-Liebig-University, Germany
Kristin Eden, Virginia Maryland College of Veterinary Medicine, USA
Daniel Ehlinger, Boston Children's Hospital/Harvard Medical School, USA
David Eisenstat, University of Alberta, Canada
Karin Eisinger, University of Pennsylvania, USA
Christine Eisner, University of British Columbia, Canada
Joel Eissenberg, Saint Louis University, USA
Stephen Ekker, Mayo Clinic, USA
Dirk Elewaut, Ghent University, Belgium
Matthew Ellis, Baylor College of Medicine, USA

Stone Elworthy, University of Sheffield, UK
Ruth Empson, University of Otago, New Zealand
Charis Eng, Lerner Research Institute Cleveland Clinic, USA
Adam Engler, University of California San Diego, USA
Ricardo Escalante, Instituto de Investigaciones Biomédicas, Spain
Kimberley Evason, Huntsman Cancer Institute, USA
Andrew Ewald, Johns Hopkins University School of Medicine, USA
Longhou Fang, Houston Methodist Research Institute, USA
Steven Farber, Carnegie Institution, USA
Henner Farin, Georg-Speyer-Haus, Germany
Mel Feany, Harvard Medical School, USA
Laura Feltri, University at Buffalo Jacobs School of Medicine and Biomedical Sciences, USA
Sarah-Maria Fendt, KU Leuven, Belgium
Hui Feng, Boston University, USA
Yi Feng, University of Edinburgh, UK
Pedro Fernandez-Fúnez, University of Minnesota, USA
Laura Ferraiuolo, University of Sheffield, UK
Miguel Ferreira, Champalimaud Foundation, Portugal
Austin Ferro, University of Minnesota, USA
Aaron Fields, University of California San Francisco, USA
David Finkelstein, Florey Institute, Australia
David Finlay, Trinity College Dublin, UK
Lydia Finley, Memorial Sloan-Kettering Cancer Center, USA
Bonnie Firestein, Rutgers, the State University of New Jersey, USA
Anthony Firulli, Indiana University School of Medicine, USA
Shannon Fisher, Boston University School of Medicine, USA
Stefanie Flunkert, QPS Austria GmbH, Austria
Riccardo Fodde, Erasmus MC, The Netherlands
Elisabeth Foerster, University of Toronto, Canada
Flavia Fontanesi, University of Miami, USA
Larry Forney, University of Idaho, USA
Jeffrey Francis, Institute of Cancer Research London, UK
J. Kimble Frazer, University of Oklahoma Health Sciences Center, USA
Christoph Freyer, Karolinska Institute, Sweden
Sylvie Friant, University of Strasbourg, France
Roland Friedel, Mount Sinai School of Medicine, USA
Niels Frimodt-Møller, Rigshospitalet, Denmark
K. Funakoshi, Yokohama City University School of Medicine, Japan
Denis Furling, Centre de Recherche en Myologie, France
Francesca Fusco, European Center for Brain Research, Italy
Rene Galindo, UT Southwestern Medical Center, USA
Michael Galko, The University of Texas MD Anderson Cancer Center, USA
Vidu Garg, Nationwide Children's Hospital, USA
Anton Gartner, The University of Dundee, UK
Daria Gavriouchkina, Okinawa Institute of Science and Technology Graduate University, Japan
Emiel Geeraerts, KU Leuven, Belguim
Robert Gendron, Memorial University of Newfoundland, Canada
Rajani George, Indiana University School of Medicine, USA
Martin Gering, The University of Nottingham, UK
Stefan Geyer, Medical University of Vienna, Austria
Angela Giangrande, IGBMC, France
Sylvain Gigout, University of Leeds, UK
Daniel Gitai, Federal University of Alagoas, Brazil
Richard Gomer, Texas A&M University, USA
Pilar Gonzalez-Cabo, University of Valencia, Spain
Martin Göpfert, University of Göttingen, Germany
Daniel Gorelick, Baylor College of Medicine, USA
Anand Goswami, Aachen University, Germany
June Goto, Cincinnati Children's Hospital Medical Center, USA
Cara Gottardi, Feinberg School of Medicine, USA

Susanne Grässel, University of Regensburg, Germany
Nicholas Greene, University of Arkansas, USA
Jorge Guerra Varela, Universidade de Santiago de Compostela, Spain
Volker Haase, Vanderbilt University School of Medicine, USA
Majid Hafezparast, University of Sussex, UK
Chris Hall, University of Auckland, New Zealand
Gianna Hammer, Duke University School of Medicine, USA
Matthias Hammerschmidt, University of Cologne, Germany
Renzhi Han, The Ohio State University Wexner Medical Center, USA
William Hancock-Cerutti, Yale University, USA
Adrian Harwood, Cardiff University, UK
Nicholas Hastie, Institute of Genetics and Molecular Medicine, Edinburgh, UK
Paul Hasty, The University of Texas Health Science Center at San Antonio, USA
Silvia Hayer, Medical University of Vienna, Austria
Fenglei He, Tulane University, USA
Miep Helfrich, University of Aberdeen, UK
Deborah Henderson, Institute of Genetic Medicine, Newcastle University, UK
Katherine Henry, University of Sheffield, UK
Jennifer Heppert, Duke University, USA
Yann Herault, IGBMC-ICS, France
Takao Hikita, Max-Plank Institute for Heart and Lung Research, Germany
Rim Hjeij, University Munster, Germany
Jorg Hohfeld, University of Bonn, Germany
Gregory Holmes, Mount Sinai School of Medicine Icahn Medical Institute, USA
Ian Hope, University of Leeds, UK
Yasmin Hough, University of Nottingham, UK
Eriola Hoxha, Neuroscience Institute Cavalieri Ottolengi, Italy
Alan Hsu, Purdue University, Bindley Bioscience Center, USA
Wei Hsu, University of Rochester Medical Center, USA
Robert Huber, Trent University, Canada
Dirk Hubmacher, Icahn School of Medicine at Mount Sinai, USA
Bruno Hudry, London Institute of Medical Sciences, UK
Billy Hudson, University of Tennessee, USA
John Hulleman, UT Southwestern Medical Center, USA
Dan Hultmark, Umea University, Sweden
Toby Hurd, IGMM, University of Edinburgh, UK
Dietmar Hutmacher, Queensland University of Technology, Australia
El Cherif Ibrahim, Aix-Marseille Université, CNRS, France
Tatsushi Igaki, Kyoto University, Japan
Andrew Intlekofer, Memorial Sloan-Kettering Cancer Center, USA
Fumitoshi Ishino, Tokyo Medical and Dental University, Japan
Angelo Iulianella, Dalhousie University, Canada
Junichi Iwata, UT Health Science Center at Houston, USA
Ethylin Jabs, Mount Sinai School of Medicine, USA
Ian Jackson, MRC Human Genetics Unit, UK
Katharina Jähn, Institut für Osteologie und Biomechanik, Germany
Rajan Jain, Perelman School of Medicine, University of Pennsylvania, USA
Gaspar Jekely, Living Systems Institute, University of Exeter, UK
Loydie Jerome-Majewska, McGill University, Canada
Jenna Jewell, University of Texas, USA
Suk-Won Jin, Yale University, USA
James Johnson, University of British Columbia, USA
Philippa Johnson, Cornell University College of Veterinary Medicine, USA
Cameron Johnstone, Olivia Newton-John Cancer Research Institute, Australia
Anne Joutel, INSERM, France
Jean-Pierre Julien, Laval University, Canada
Monica Justice, Hospital for Sick Children, Canada
Patricia Kabitzke, PsychoGenics, Inc, USA
Daniel Kaganovich, Hebrew University of Jerusalem, Israel
Philipp Kahle, University of Tübingen, Germany

Sharanya Kalasekar, Huntsman Cancer Institute, University of Utah, USA
Rita Kandel, Mount Sinai Hospital, Canada
Min Ji Kang, University of Ulsan College of Medicine, Republic of Korea
Nagarajan Kannan, Mayo Clinic, USA
Peter Kannu, Hospital for Sick Children, Canada
Mike Karl, University of Washington, USA
Seema Kaushalya Tiwari-Woodruff, School of Medicine, University of California Riverside, USA
Jonathan Kelber, California State University, Northridge, USA
Lisa Kelly, University of California Berkeley, USA
Maurice Kernan, Stony Brook University, USA
Dilan Khalili, The Wenner-Gren Institute, Sweden
Salman Khetani, University of Illinois at Chicago, USA
Robin Kimmel, University of Innsbruck, Austria
Veronica Kinsler, UCL Institute of Child Health, UK
Janine Kirby, University of Sheffield, UK
Kassandra Kisler, University of Southern California, USA
Toshihiro Kitamoto, University of Iowa, USA
Arnaud Klein, UMRS 974 UPMC - INSERM - AIM, France
Karl-Heinz Klempnauer, University of Münster, Germany
Shantha Kodihalli, Emergent BioSolution, Canada
David Kokel, University of California San Francisco, USA
Stephen Konieczny, Purdue University, USA
Eiki Koyama, Children's Hospital of Philadelphia (CHOP), USA
Deborah Krakow, University of California Los Angeles, USA
David Krantz, University of California Los Angeles, USA
Harm Krugers, University of Amsterdam, The Netherlands
Takayuki Kuraishi, Kanazawa University, Japan
Yeshwant Kurhe, Rush University Medical Center, India
Veronika Kuscha, Technische Universitat Dresden, Germany
Zahra Labbaf, Universitat Munster, Germany
Jenni Lahtela, Institute for Molecular Medicine, Finland
Angela Laird, Macquarie University, Australia
Robert Lalonde, Université de Rouen, France
Jennifer Lamberts, Ferris State University, USA
Ryan Lamont, University of Calgary, USA
Dirk Lange, University of British Columbia, Canada
David Langenau, Massachusetts General Hospital, USA
Collette LaVigne, UT SouthWestern Medical Center, USA
Michael Lawrence, Baylor Scott & White Research Institute, USA
Joost le Feber, University of Twente, The Netherlands
Jiyoung Lee, Medical Research Institute, Tokyo, Japan
Simon Leedham, Wellcome Trust Centre for Human Genetics, UK
Stylianos Lefkopoulos, Max-Plank Institute of Immunobiology and Epigenetics, Germany
Michelle Letarte, University of Toronto, Canada
Mark Lewandoski, NCI, USA
Jada Lewis, University of Florida, USA
Bo Li, Zhongshan School of Medicine, China
Min Li, The University of Oklahoma Health Sciences Center, USA
Xue-Jun Li, University of Illinois, USA
Ellen Lien, University of Southern California, USA
Soren Lienkamp, University of Freiburg, Germany
Hui-Ying Lim, University of Oklahoma, USA
Joy Lincoln, Nationwide Children's Hospital, USA
Chunqiao Liu, Sun Yat-Sen University, China
Karen Liu, King's College London, UK
Cecilia Lo, University of Pittsburgh, USA
Adolfo Lopez de Munain, BioDonostia, Spain
Philip LoVerde, UT Health San Antonio, USA
Martin Lowe, University of Manchester, UK
Miodrag Lukic, University of Kragujevac, Serbia

Laura Machesky, Beetson Institute, University of Glasgow, UK
Cressida Madigan, University of California, San Diego, USA
Keith Maggert, University of Arizona, USA
Marcello Maggiolini, University of Calabria, Italy
Donna Maglott, NCBI, USA
Virginie Mansuy-Aubert, Loyola University, USA
Denise Marciano, UT SouthWestern Medical Center, USA
Maria Marco, University of California Davis, USA
Melanie Mark, Ruhr-University Bochum, Germany
Jeanne Marrazzo, University of Alabama at Birmingham, USA
Francisco Martin, GENYO, Spain
Paul Martin, University of Bristol, UK
Alfonso Martín-Peña, University of Florida, USA
Valentina Massa, University of Milan, Italy
Ivica Matak, University of Zagreb, Croatia
Saumi Mathews, University of Nebraska Medical Center, USA
Aaron Maule, Queens University, Belfast, UK
Robert Maxson, USC Keck School of Medicine, USA
Ulrike Mayer, University of East Anglia, UK
Thomas Maynard, The George Washington University, USA
Thibault Mayor, University of British Columbia, Canada
Erin Mcallum, Florey Institute of Neuroscience and Mental Health, Australia
Jessica McCann, Duke University School of Medicine, USA
Glenn McConkey, University of Leeds, UK
Alicia McConnell, University of East Anglia, UK
Rebecca McGreal, Albert Einstein College of Medicine, USA
Chantelle McIntyre, University of Adelaide, Australia
Cynthia McMurray, Berkeley Lab, USA
Kelly M. McNagny, University of British Columbia, Canada
Kayla Meade, Midwestern State University, USA
Ashish Mehta, Victor Chang Cardiac Research Institute, Australia
Philipp Mergenthaler, Charité Universitätsmedizin Berlin, Germany
Pablo Meyer, IBM, USA
Craig Micchelli, Washington University School of Medicine, USA
Fabrizio Michetti, Universita Cattolica del Sacro Cuore, Italy
Alison Michie, University of Glasgow, UK
Irene Miguel-Aliaga, Imperial College London, UK
Marco Milan, IRB Barcelona, Spain
Rachel Miller, McGovern Medical School, USA
Marina Mione, University of Trento, Italy
Mila Mirceta, University of Toronto, Canada
Christopher Mirchell, University of Ulster, UK
Zhasmine Mirzoyan, University of Milan, Italy
Yuji Mishina, University of Michigan, USA
Biswapriya Misra, Wake Forest School of Medicine, USA
Thimios Mitsiadis, Universität Zürich, Switzerland
Cecilia Moens, Fred Hutchinson Cancer Research Center, USA
Alexander Moise, Laurentian University, Canada
Cesare Montecucco, University of Padova, Italy
Lieve Moons, KU Leuven, Belgium
Mariya Moosajee, University College London, UK
Danielle Mor, Childrens Hospital of Philadelphia - Research Institute, USA
Paula Moran, University of Nottingham, UK
Noemi Morello, Universiti di Toronto, Italy
Alan Morgan, University of Liverpool, UK
Nuria Morral, Indiana University School of Medicine, USA
Jennifer Morton, The Beatson Institute, UK
Christian Mosimann, University of Zurich, Switzerland
Tomas Mracek, Institute of Physiology CAS, Czech Republic
Marc Muller, University of Liège, Belgium

Coleen Murphy, Princeton University, USA
Geoffrey Murphy, University of Michigan, USA
Peter Murray, Max Planck Insitute, Germany
Francis Mussai, University of Birmingham, UK
Laura Musselman, Binghamton University, USA
Sandesh Nagamani, Baylor College of Medicine, USA
Istvan Nagy, Imperial College London, UK
Lazlo Nagy, University of Debrecen, Hungary
Norimasa Nakamura, Osaka University, Japan
Masanori Nakayama, Max Planck Institute for Heart and Lung Research, Germany
Juan Antonio Navarro, University of Regensburg, Germany
Aaron Neiman, Stony Brook University, USA
Michel Ney, Center of Research in Myology, France
Dominic Ng, University of Toronto, Canada
Hoang Nguyen, Baylor College of Medicine, USA
Teresa Niccoli, University College London, UK
Beatrice Nico, University of Bari, Italy
Stefania Nicoli, Yale School of Medicine, USA
Brian Nieman, Hospital for Sick Children, Canada
Philipp Niethammer, Memorial Sloan-Kettering Cancer Center, USA
Jeffrey Noebels, Baylor College of Medicine, USA
Patrick Nolan, MRC Harwell, UK
Trista North, Harvard Medical School, USA
Lauryl Nutter, The Centre for Phenogenomics, Canada
Tomoko Obara, University of Oklahoma Health Sciences Center Biomedical Research Center, USA
Johannes Oberwinkler, University of Marburg, Germany
John O'Brien, UT-Houston Health Science Center, USA
Stefan Oehlers, Centenary Institute, Australia
Phil Oel, University of Alberta, Canada
William Okech, University of Pittsburgh, USA
Louise O'Keefe, University of Adelaide, Australia
Ceri Oldreive, University of Birmingham, UK
Andrew Olive, Michigan State University, USA
Liam O'Mahony, University of Zurich, Switzerland
Heymut Omran, Department of Pediatrics, University Hospital Muenster, Germany
Ferenc Orosz, Institute of Enzymology, Hungarian Academy of Sciences, Hungary
Isabel Orriss, Royal Veterinary College, University of London, UK
Larry Ostrowski, Marsico Lung Institute, USA
Michael Pack, University of Pennsylvania, USA
Antonio Pagán, Cambridge University, UK
Michael Palladino, University of Pittsburgh, USA
John Parant, University of Alabama at Birmingham, USA
Gilberto Pardo-Andreu, Universidad de La Habana, Cuba
David Parichy, University of Virginia, USA
Sung Wook Park, Seoul National University College of Medicine, Republic of Korea
Andreas Patsalos, Johns Hopkins University, USA
Kessen Patten, INRS Institute Armand-Frappier, Canada
Ostrowski Patten, INRS Institute Armand-Frappier, Canada
Liz Patton, Edinburgh University, UK
Gregory Payne, UCLA School of Medicine, USA
David Pearce, Sanford Research/USD, USA
Catherine Pears, University of Oxford, UK
Helen Pearson, Cardiff University, UK
Hui Peng, UT Southwestern Medical Center, USA
Jeroen Pennings, National Institute for Public Health and the Environment (RIVM), The Netherlands
Brian Perkins, Cleveland Clinic, USA
Muriel Perron, Université Paris Sud, France
Toby Phesse, Cardiff University, UK
Dana Philpott, The Hospital for Sick Children, Canada
Lucie Picchio, Université Clermont Auvergne, France

Marita Pietrucha-Dutczak, Medical University of Silesia, Poland
Andrew Pitsillides, Royal Veterinary College, UK
Ross Poche, Baylor College of Medicine, USA
Katerina Politi, Memorial Sloan Kettering Cancer Center, USA
Larisa Poluektova, University of Nebraska Medical Center, USA
Mark Pook, Brunel University London, UK
Cristina Porcheri, University of Zurich, Switzerland
Vittorio Porciatti, University of Miami, USA
George Prendergast, Lankenau Institute for Medical Research, USA
Xavier Prieur, University Nantes, France
Catrin Pritchard, University of Leicester, UK
Sergey Prykhozhij, Max-Plank Institute for Molecular Genetics, Germany
Nathalie Pujol, Centre d'Immunologie de Marseille-Luminy, France
Vinothkumar Rajan, Dalhousie University, Berman Zebrafish Lab, USA
Anjana Ramdas Nair, New York University Abu Dhabi, Abu Dhabi
Francesco Ramirez, Icahn School of Medicine at Mount Sinai, New York, USA
Anusha Ratneswaran, University of Western Ontario, Canada
John Rawls, University of North Carolina School of Medicine, USA
Sarah Rea, Harry Perkins Institute of Medical Research, Australia
Michael Redd, University of Utah, USA
Roger Reeves, Johns Hopkins University School of Medicine, USA
Evan Reid, Cambridge Institute for Medical Research, UK
Ralf Reilmann, Universitaetsklinikum Muenster, Germany
Dieter Reindhardt, McGill University School of Medicine, Canada
Flávio Reis, Faculty of Medicine - University of Coimbra, Portugal
Michael Rera, Sorbonne-Université, France
Sang Rhee, Oakland University, USA
Saima Riazuddin, University of Maryland, USA
Angeles Ribera, University of Colorado at Anschutz Medical Center, USA
Emma Ristori, Yale University, USA
Jennifer Ritchie, University of Surrey, UK
Mark Rizzo, University of Pittsburgh, USA
Regina Rodrigo, Instituto de Investigación Sanitaria La Fe, Spain
Thomas Roeder, Kiel University, Germany
Minna Roh-Johnson, University of Utah, USA
Beth Roman, University of Pittsburgh, USA
Randall Roper, Indiana University, Purdue School of Science, USA
David Rosen, Washington University School of Medicine in St. Louis, USA
Katharina Rosenbusch, University of Groningen, The Netherlands
Fabio Rossi, University of British Columbia, Canada
Leonardo Rossi, University of Pisa, Italy
Matthieu Roustit, University Grenoble Alpes, France
Jan Rozman, Helmholtz Zentrum München, Germany
Speranza Rubattu, Istituto Neurologico Mediterraneo Neuromed, Italy
James Russell, University of Maryland School of Medicine, USA
Thomas Rutkowski, The University of Iowa, USA
Frank Rutsch, Muenster University Children's Hospital, Germany
Aimee Ryan, McGill University, Canada
Alvaro Sagasti, University of California Los Angeles, USA
Yukio Saijoh, University of Utah, USA
Lynn Sakai, Oregon Health & Science University and Shriners Hospital for Children, USA
Rodney Samaco, Baylor College of Medicine, USA
Valerie Sampson, Alfred I. duPont Hospital for Children, USA
Berta Sanchez-Laorden, University of Murcia, Spain
Marco Sandri, Venetian Institute of Molecular Medicine, Italy
Owen Sansom, The Beatson Institute for Cancer Research, UK
Matthew Sapio, National Institutes of Health, USA
Marco Sardiello, Baylor College of Medicine, USA
Emiko Sato, Tohoku University, Japan
Smita Saxena, University of Bern, Switzerland

Amnon Schlegel, University of Utah School of Medicine, USA
Miriam Schmidts, University Freiburg Medical Center, Germany
Frank Schnorrer, Institut de Biologie du Developpement de Marseille, France
Markus Schober, New York University, USA
Paul Schofield, University of Cambridge, UK
Stephanie Schorge, University College London, UK
Oren Schuldiner, Weizmann Institute of Science, Israel
Stefan Schulte-Merker, Hubrecht Institute (KNAW), The Netherlands
Daryl Scott, Baylor College of Medicine, USA
Patrick Seed, Northwestern University Feinberg School of Medicine, USA
Carolyn Sevier, Cornell University College of Veterinary Medicine, USA
Celia Shiau, University of North Carolina at Chapel Hill, USA
Daesung Shin, University of Buffalo, Hunter James Kelly Research Institute, USA
Donghun Shin, University of Pittsburgh, USA
Ody Sibon, University of Groningen, The Netherlands
Detlef Siemen, University of Magdeburg, Germany
Marina Simian, Instituto de Nanosistemas, Universidad Nacional de San Martín, Argentina
Peter Siska, Univeristy Hospital Regensburg, Germany
Karim Si-Tayeb, L'institut du Thorax, UMR INSERM1087/CNRS6291, IRS-UN, France
Paris Skourides, University of Cyprus, Cyprus
Søs Skovsø, University of British Columbia, Canada
Amy Skubitz, University of Minnesota, USA
William Sly, Saint Louis University School of Medicine, USA
Ian Smyth, Monash University, Australia
Elizabeth Sockett, University of Nottingham, UK
Mozhdeh Sojoodi, Massachusetts General Hospital, USA
Michele Solimena, Dresden University of Technology, Germany
Ferdinando Squitieri, Mendel Institute of Human Genetics, Italy
Jemeen Sreedharan, King's College London, UK
David Stachura, California State University, Chico, USA
Kryn Stankunas, University of Oregon, USA
Chloe Stanton, Medical Research Council Human Genetics Unit, UK
Thaddeus Stappenbeck, Washington University School of Medicine in St. Louis, USA
Michelle Starz-Gaiano, University of Maryland Baltimore County, USA
Jenna Steinle, Wayne State University, USA
Rodney Stewart, University of Utah, USA
Catherine Stothard, Newcastle University, UK
Warren Strober, National Institutes of Health, USA
Sunil Sudarshan, University of Alabama at Birmingham, USA
Margaret Man-Ger Sun, University of Western Ontario, Canada
Kate Sutherland, Walter and Eliza Hall Institute, Australia
Amanda Swain, Instititute of Cancer Research, UK
Istvan Szokodi, University of Pecs, Hungary
Jacqueline Tabler, Max Planck Institute of Molecular Cell Biology and Genetics, Germany
Jordi Tamarit, Lleda University, Spain
Shumin Tan, Tufts University School of Medicine, USA
Teruyuki Tanaka, University of Tokyo, Japan
Simon Tang, Washington University in St Louis, USA
Javad Tavakoli, Flinders University, Australia
Filippo Tempia, University of Turin, Italy
Vinay Tergaonkar, ASTAR Singapore, Singapore
Michael Themis, Brunel University, UK
Ulrich Theopold, Stockholm University, Sweden
Thimmasettappa (Swamy) Thippeswamy, Iowa State University, USA
Tim Thomas, Walter and Eliza Hall Institute of Medical Research, Australia
Barry Thompson, Crick Institute, UK
David Thompson, Purdue University, USA
Leslie M. Thompson, UC Irvine, USA
Eugenia Thrushina, Mayo Clinic, USA
Glen Tibbits, Simon Fraser University, Canada

Paul Timpson, The Garvan Institute of Medical Research, Australia
David Tobin, Duke University Medical Center, USA
Giang Tong, Deutsches Herzzentrum Berlin, Germany
Qingchun Tong, University of Texas, USA
Carmel Toomes, University of Leeds, UK
Dan Tracey, Indiana University Bloomington, USA
Lauren Tracey, The Hospital for Sick Children, Canada
Andreas Traweger, Paracelsus Medical University, Austria
Sunny Trivedi, Stanford University, USA
Eirini Trompouki, MPI, Germany
Li-Huei Tsai, MIT, USA
Abigail Tucker, King's College London, UK
Tully Underhill, University of British Columbia, Canada
Christian Ungermann, University of Osnabrück, Germany
Anthony Uren, Imperial College London, UK
Ludovic Vallier, Cambridge Stem Cell Institute and the Wellcome Sanger Institute, UK
Bart van de Sluis, University of Groningen, The Netherlands
Maaike van den Berg, University of Bristol, UK
Patricija van Oosten-Hawle, University of Leeds, UK
Julien Vermot, IGBMC, France
Katarina Vukojevic, University of Split, Croatia
Richard Wade-Martins, University of Oxford, UK
John Wallingford, University of Texas at Austin, USA
Lucas Waltzer, Universite Clermont Auvergne, CNRS, France
Jingjing Wang, University of New Mexico, USA
Tao Wang, National Institute of Biological Sciences, China
Michael Wangler, Baylor College of Medicine, USA
Alastair Watson, University of East Anglia, UK
Alanna Watt, McGill University, Canada
Joshua Wechsler, Feinburg School of Medicine Northwestern, USA
Wei Wei, BC Cancer, Canada
Hans Weiher, Hochschule Bonn-Rhein-Sieg, Germany
Rong Wen, University of Miami, School of Medicine, USA
Wolfgang Weninger, Medical University of Vienna, Austria
Richard White, London School of Hygiene and Tropical Medicine, UK
Robin Williams, Royal Holloway, UK
Trevor Williams, University of Colorado Denver, USA
Meredith Wilson, Carnegie Institution, Washington, USA
Rebecca Wingert, University of Notre Dame, USA
Luke Wiseman, Scripps Research Institute, USA
Jonathan Wood, University of Sheffield, UK
Gavin Woodhall, Aston University, UK
Anna Wredenberg, Karolinska Institute, Sweden
Anping Xia, Stanford University, USA
Ying Xia, University of Cincinnati, USA
Heping Xu, Queen University Belfast School of Medicine, Ireland
Michael Yaffe, University of California San Diego, USA
Shinya Yamamoto, Baylor College of Medicine, USA
Shu Yang, NIH, USA
Makiko Yasuda, Icahn School of Medicine Mount Sinai, USA
Bing Ye, University of Michigan, USA
Mark Yorek, University of Iowa, USA
Matthew Young, National Cancer Institute, USA
Min Yu, University of Southern California, USA
Y. Eugene Yu, Roswell Park Cancer Institute, USA
Catherine Yzydorczyk, Lausanne University Hospital, Switzerland
Stephane Zaffran, Aix-Marseille University, France
Frank Zaucke, Orthopaedic University Hospital Friedrichsheim in Frankfurt/Main, Germany
Stefan Zauscher, Duke University, USA
Andrew Zelhof, Indiana University, USA

Zhiqiang Zeng, University of Edinburgh, UK
Qingjiong Zhang, Sun Yat-Sen University, China
Sheng Zhang, University of Texas, USA
Lei Zhao, Shandong Provincial Cancer Hospital and Institute, China
Qing Yin Zheng, Case Western University, USA
Wei Zheng, National Center for Advancing Translational Sciences (NCATS), NIH, USA
Jingsong Zhou, University of Texas at Arlington, USA
Yang Zhou, Brown University, USA
Teresa Zimmers, Indiana University, USA
Leonard Zon, Harvard Stem Cell Institute, USA
Robert Zweigerdt, Hannover Medical School, Germany
